# Rice Stripe Mosaic Disease: Characteristics and Control Strategies

**DOI:** 10.3389/fmicb.2021.715223

**Published:** 2021-07-29

**Authors:** Zhiyi Wang, Biao Chen, Tong Zhang, Guohui Zhou, Xin Yang

**Affiliations:** Guangdong Province Key Laboratory of Microbial Signals and Disease Control, College of Plant Protection, South China Agricultural University, Guangzhou, China

**Keywords:** rice stripe mosaic virus, rice disease, Rhabdovirus, *Recilia dorsalis*, disease control

## Abstract

Rice stripe mosaic disease (RSMD) is caused by the *rice stripe mosaic virus* (RSMV; genus *Cytorhabdovirus*, family *Rhabdoviridae*). In recent years, significant progress has been made in understanding several aspects of the disease, especially its geographical distribution, symptoms, vectors, gene functions, and control measures. Since RSMD was first detected in southern China in 2015, it has been found in more and more rice growing areas and has become one of the most important rice diseases in southern China. RSMV is transmitted by the leafhopper *Recilia dorsalis* in a persistent-propagative manner, inducing yellow stripes, a slight distortion of leaves, increased tillers, and empty grains in rice plants. The virus has a negative-sense single-strand RNA genome of about 12.7 kb that encodes seven proteins: N, P, P3, M, G, P6, and L. Several molecular and serological tests have been developed to detect RSMV in plants and insects. The disease cycle can be described as follows: RSMV and its vector overwinter in infected plants; viruliferous *R. dorsalis* adults transmit the virus to spring rice and lay eggs on the infected seedlings; the next generation of *R. dorsalis* propagate on infected seedlings, become viruliferous, disperse, and cause new disease outbreaks. Control measures include monitoring and accurate forecasting, selecting disease-resistant varieties, improving cultivation systems, covering rice seedling nurseries with insect-proof nets, and using pesticides rationally. Inappropriate cultivation systems, pesticide overuse, and climatic conditions contribute to epidemics by affecting the development of vector insects and their population dynamics.

## Introduction

In 2015, rice stripe mosaic disease (RSMD) was first discovered in the southwestern rice region of Guangdong province in China. The disease is caused by rice stripe mosaic virus (RSMV) and transmitted by the leafhopper *Recilia dorsalis* (Yang et al., 2017a,b; Kuhn et al., 2020), and mainly occurs in seven provinces of southern China (Chen et al., 2019a). Significant progress has been made in recent years in understanding this disease. Therefore, this review aims to summarize recent advances in our knowledge of its geographical distribution, host range and symptomatology, transmission biology, influence on vectors, genome organization, gene functions, diagnostic tests, disease management, and factors that contribute to intermittent outbreaks.

## Geographical Distribution

**Figure 1:**
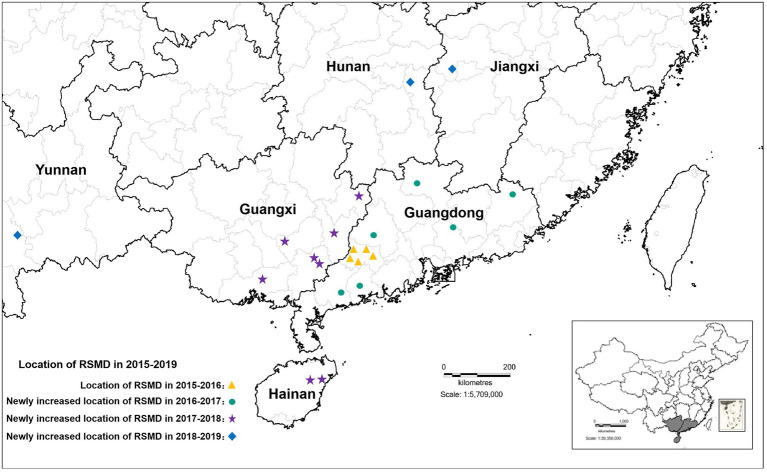
The distribution of rice stripe mosaic virus (RSMV) in South China from 2015 to 2019 modified from Chen et al. (2019a).

From 2015 to 2016, RSMD only occurred in southwestern Guangdong in southern China; Luoding rice areas had the most serious occurrence, with a 70% field incidence. From 2016 to 2018, the disease gradually spread throughout southern China, and diseased plants were found in eight rice-growing areas in Guangdong province (Yunfu, Maoming, Zhanjiang, Yangjiang, Huizhou, Heyuan, Shaoguan, and Meizhou), four rice-growing areas in Guangxi province (Wuzhou, Hezhou, Yulin, and Qinzhou), and two rice-growing areas Hainan province (Ding’an and Tunchang County; Yang et al., 2018). Among them, Yunfu city in Guangdong province was the most affected, with 165 fields under investigation. Diseased plants were found in nearly 75.16% of fields, and the incidence rate was up to 60%. The second most affected city was Wuzhou in Guangxi province, with 34 fields under investigation; diseased plants were found in about 61.76% of fields. This disease was only occasionally found in other rice areas (Chen et al., 2019a). From 2019 to now, RSMV-infected rice plants have also occasionally been observed in parts of Jiangxi, Hunan, and Yunnan province (Figure 1), indicating that the distribution of RSMD is gradually expanding and that the risk of harm to rice production is increasing.

## Host Range and Symptomatology

In nature, the host plants of RSMV include rice, *Digitaria sanguinalis*, *Monochoria vaginalis*, *Eleusine indica*, *Alopecurus aequalis*, and several other gramineous weeds. Meanwhile, tobacco, maize, and *Arabidopsis thaliana* cannot be infected by RSMV under greenhouse conditions.

**Figure 2:**
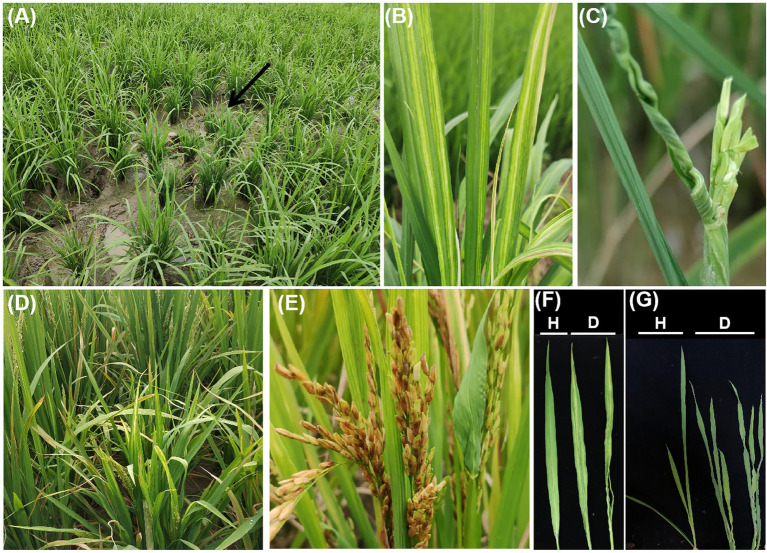
Symptoms of RSMV-infected rice plants. **(A)** Diseased plants in the field, indicated by a black arrow. **(B)** Mosaic stripes on leaf. **(C)** Twisting of RSMV-infected rice leaves. **(D)** Plant dwarfing. **(E)** Incomplete heading. **(F,G)** Mosaic and twisting characteristics compared with RSMV-free leaves. Healthy leaves are marked with an H, and diseased leaves are marked with a D.

Susceptible rice plants are mainly characterized by slight dwarfing, the presence of twisted leaves exhibiting striped mosaicism, an increased number of tillers, inferior heading, and mostly unfilled grains (Yang et al., 2017a; Chen et al., 2019a; Figure 2). Rice is susceptible to RSMV during all growth stages, but the symptoms depend on the growth stage at the time of infection. Plants infected before the three-leaf stage show dwarfing, yellow stripes, mosaicking, inward-curling leaves, excessive tillering, and failing to head. Plants infected at the early tillering stage show slight symptoms, resulting in dwarfing, leaves with partial stripes and mosaics, slightly increased tiller numbers, and unfilled grains. Plants infected at the late tillering stage show no visible symptoms and can head normally. In addition, plants infected in warmer environments show obvious striped mosaic symptoms and their leaves may be severely deformed and curled. Severely affected fields typically show obvious disease centers; diseased plants appear alternately with healthy plants and are distributed mainly near or at field edges (Chen et al., 2019a).

Different rice varieties exhibit slightly different symptoms. RSMV afflicts hybrid rice more than inbred rice, as indicated by field surveys (Chen et al., 2019a). RSMV-infected hybrid rice plants show dwarfing; their leaves show bright yellow or light yellow at the beginning and then appear as green and yellow stripes, which gradually extend to the middle of the blade. Finally, the whole leaf appears to have mosaics. Most *indica* and *japonica* rice infected with RSMV show a slight dwarfism with slight stripe mosaics on the leaves (Chen et al., 2019a). Moreover, three RSMV-infected cultivated rice varieties representing *indica* (cv. Meixiangzhan), hybrid *indica* (cv. Wuyou1179), and *japonica* (cv. Nipponbare) rice all exhibited typical symptoms, but slight differences in symptoms and yield loss were observed, with Meixiangzhan showing the most severe symptoms and yield reduction (Chen et al., 2019b).

## Transmission Biology

Rice stripe mosaic virus is transmitted by *R. dorsalis* in a persistent-propagative manner, but it cannot be transmitted mechanically, vertically, or by seeds. Under laboratory conditions, it was found that *R. dorsalis* transmit the virus with a 57.1% efficiency (Yang et al., 2017b). *Nephotettix virescens* can also acquire the virus under laboratory conditions and the transmission efficiency is only 10% (Zhao et al., 2019), so the possibility that *N. virescens* serves as a natural vector of RSMV requires further confirmation.

Both *R. dorsalis* nymphs and adults can transmit RSMV, and nymphs have a higher transmission efficiency. After a minimum acquisition access period of 3 min, the RSMV acquisition rates of nymphs and adults were 24.4 and 19.2%, respectively. The acquisition rate gradually increased as the feeding time increased, and after 3 h of feeding, the rates in nymphs and adults were increased to 66.7 and 58.9%, respectively. Virus-positive *R. dorsalis* could transmit RSMV to healthy plants after a minimum feeding period of 30 min, and after 1 h of feeding in infected plants, the transmission rate in RSMV-infected nymphs and adults reached 57.1 and 50.0%, respectively (Yang et al., 2017b). The circulative transmission period of RSMV in most *R. dorsalis* specimens was 8–16 days (Yang et al., 2017b). Around 12 days after the first access to diseased plants, RSMV spread to all organs of most *R. dorsalis* specimens (Zhao et al., 2019). Most individuals could continuously transmit RSMV throughout their lives, some intermittently transmitted the virus at intervals of 2–6 days, and a few could not transmit the virus at any time during their lifespan (Yang et al., 2017b).

## Influence on Vector Insect

When *R. dorsalis* specimens were infected with RSMV, viruliferous nymph adulteration was prolonged and the survival rate, adult emergence rate, and egg hatching rate were reduced compared with those of nonviruliferous *R. dorsalis*. In addition, viruliferous *R. dorsalis* adults preferred RSMV-free rice plants, whereas nonviruliferous *R. dorsalis* adults preferred RSMV-infected rice plants, indicating that RSMV affects the host selectivity of *R. dorsalis* (Li et al., 2020). On the other hand, viruliferous *R. dorsalis* fed on healthy rice plants at a greater frequency and for a longer time than nonviruliferous leafhoppers, thereby improving the RSMV transmission efficiency (Li et al., 2020). This kind of impact on host selection preference and vector feeding behavior has also been reported in other plant viruses, such as southern rice black-streaked dwarf virus (Wang et al., 2013) and cucumber mosaic virus (CMV; Mauck et al., 2010). It is speculated that, over long-term evolution, viruses have improved their self-transmission efficiency by regulating their host selection preference and vector feeding behavior.

## Virion Composition and Morphology

Rice stripe mosaic virus is a new species in the genus *Cytorhabdovirus*, family *Rhabdoviridae* (Yang et al., 2017a; Kuhn et al., 2020). It has similar structural and biological features to other plant viruses that belong to the *Rhabdoviridae* family. Basically, plant rhabdoviruses are composed of an infectious nucleocapsid and an outer phospholipid envelope layer. The nucleocapsid is a ribonucleoprotein (RNP) complex that consists of the genomic RNA (gRNA), nucleoprotein (N), phosphoprotein (P), and a large polymerase protein (L). The RNP is coated by matrix protein (M) and the M protein is wrapped in an outer layer that contains spike-like surface projections composed of glycoprotein (G; Jackson et al., 2005; Dietzgen et al., 2017).

**Figure 3:**
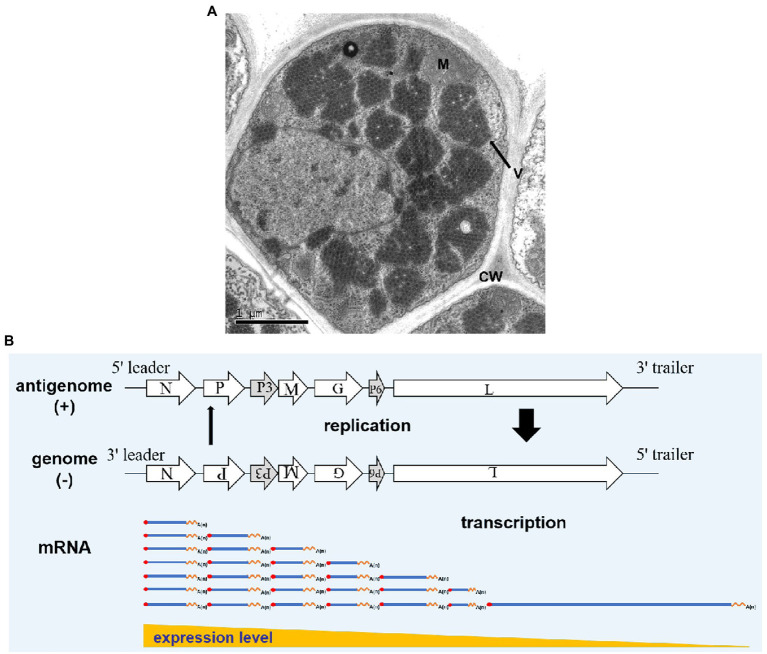
Rice stripe mosaic virus virion morphology and schematic diagram of its transcription and replication. **(A)** Transmission electron micrograph of RSMV in an infected rice cell. V represents virions, M represents a mitochondrion, and CW represents the cell wall. **(B)** Schematic diagram of RSMV vRNA transcription and replication modified from Wagner and Jackson (1997) and Jackson and Li (2016). mRNA is transcribed from genomic RNA (gRNA) in the concentrations N > P > P3 > M > G > P6 > L. As N, P, and L proteins accumulate in abundance, antigenomic RNA (agRNA) replication is initiated, followed by gRNA replication. Thick and thin arrows indicate that the accumulation of agRNA is lower than that of genomic RNA.

In RSMV-infected rice leaves, mature RSMV virions are enveloped in bacilliforms that are 300–375 nm long and 45–55 nm wide. RSMV virions always accumulate in the cytoplasm and form large numbers of crystalline structures that occupy nearly the entire cytoplasmic space, while some virions also gather in the vesicles or around the nucleus (Figure 3; Yang et al., 2017a; Liu et al., 2019b). In RSMV-infected *R. dorsalis*, RSMV virions are present in enveloped and non-enveloped forms and distributed in different tissues (Zhao et al., 2019). Most virions have an average length of 325 nm and a width of 50 nm (Yang et al., 2017b).

## Viral Genomic Structure and Genetic Variation

Rice stripe mosaic virus genome is a negative-sense single-stranded RNA with a full length of 12,774 nt. The complementary sense RNA of RSMV contains seven non-overlapping open reading frames (ORFs). The ORFs and 3ꞌ–5ꞌ non-coding regions of RSMV have similar features to other rhabdoviruses, containing three conserved gene regulatory elements (the transcription termination region, the gene junction region, and the transcription initiation region), and the 3ꞌ leader to 5ꞌ trailer non-coding regions have partially complementary nucleotide sequences that can form a pot handle-like structure (Jackson et al., 2005; Yang et al., 2017a). Currently, 13 RSMV isolates from the Guangdong, Guangxi, and Hainan provinces can be found in the GenBank database. Sequence analysis showed that the nucleotide and amino acid sequences of 13 RSMV isolates were highly conserved and shared 99.4–100% and 98.9–100% identity, respectively (Yang et al., 2018). RSMV isolates from all over Guangdong share a lower nucleotide identity between them than isolates from Guangxi and Hainan, suggesting that the virus may have originated in Guangdong (Yang et al., 2018).

Studies have shown that mutations in some virus amino acid sequences can affect the epidemic distribution of their associated diseases. For example, the E1 and E2 proteins encoded by Chikungunya Virus (CHIKV) have A226V and L210Q amino acid mutations, respectively. The L210Q mutation enhances CHIKV to infect *Aedes albopictus* and promotes the dispersal of this disease (Tsetsarkin et al., 2007; Tsetsarkin and Weaver, 2011). The A982V mutation of the Zika virus (ZIKV) NS1 protein enhances the infection of its vector, potentially promoting disease epidemics (Xia et al., 2018). Likewise, the ZIKV NS5 protein has a mutation between M and V at the 2,634 amino acid position that plays an important role in the outbreak of ZIKV (Liu et al., 2019a). Furthermore, an amino acid I440T mutation in the structural protein C/prM/E encoded by the Dengue virus-I may have played an important role in its outbreak in 2017 in Xishuangbanna, China (Lin et al., 2019). RSMV isolates also have specific amino acid mutations on the L protein (Yang et al., 2018), suggesting that certain genetic variations may have occurred during its geographic spread, which in turn promoted the spread of the virus in specific areas.

## Function of Rsmv-Encoded Proteins

Rice stripe mosaic virus sequentially encodes seven proteins (P1–P7), including five structural proteins (P1, P2, P4, P5, and P7) and two non-structural proteins (P3 and P6; Table 1). Among the structural proteins, the P1 protein is predicted to be the N protein that contains two putative nuclear localization signals (NLSs) at the amino (14–45 aa) and carboxy (440–474 aa) termini (Yang et al., 2017a). The rhabdovirus N protein is highly conserved, and it was reported to regulate the transcription and replication of the genome by affecting the recognition process of transcription signals, and it can also induce host immune response (Luo et al., 2007). In addition, the N protein can tightly bind to viral gRNA to prevent its cleavage by host cell nucleases (Luo et al., 2007). The P2 protein of RSMV is predicted to be the P protein. Although, its amino acid sequence shows a low identity to the P proteins of other rhabdoviruses, it has similar multiple phosphorylation sites to others (Yang et al., 2017a). The P protein of rhabdoviruses serves as a cofactor for the viral polymerase and mainly mediates the correct positioning and connection of the L protein on the N-RNA template. During the synthesis of N protein, P protein can also act as a molecular chaperone to prevent N protein self-aggregation by forming N–P complexes to bind genomic RNA (Barik and Banerjee, 1992; Delmas et al., 2010; Zhou et al., 2019a; Zhang et al., 2020b). The P protein of alfalfa dwarf virus (ADV) and lettuce necrotic yellows virus (LYNV) were reported to be RNA silencing suppressors (Mann et al., 2015; Bejerman et al., 2016). In addition, the P protein of barley yellow striate mosaic virus (BYSMV) can interact with the RNA degrading factor CCR4 (carbon catabolite repression 4) to change its location. Carbon catabolite repression 4 (CCR4) also non-specifically degrades the poly A of host mRNA, increases the specific binding efficiency of the N protein and the genome, and promotes viral replication in the host (Gao et al., 2020). The P4 protein of RSMV is predicted to be the M protein, which is a weak silencing suppressor. It can interact with silencing gene 3 (rice endogenous suppressor) and can affect host antiviral RNA silencing functions (Zhang et al., 2020a). The overexpression of the RSMV M protein in rice induces symptoms such as dwarfing, increased tiller number, and growth retardation, indicating that M may participate in the production of virus-induced symptoms (Zhang et al., 2020a). The M protein of RSMV can also interact with the complement control protein (CCP) region of the *R. dorsalis Hig* gene (Hikaru genki, neuron specific factor) and can participate in the vector’s antiviral processes (Wang et al., 2020). In addition, RSM M protein can interact with the jasmonic acid signaling pathway and inhibit host’s resistance function (Li et al., 2021). Furthermore, the rhabdovirus M protein is an internal component of the virion; participates in virion assembly; and promotes the budding process of rhabdoviruses, the expression of viral genes, and their transport in the nucleus and cytoplasm (Harty et al., 1999; Sun et al., 2018). In addition, the M protein of SYNV can induce superinfection exclusion (SIE) and prevent secondary infections (Zhou et al., 2019b). The P5 protein of RSMV is predicted to be the G protein; it has a signal peptide and seven glycosylation sites at the N terminus and a transmembrane region at the C terminus (Yang et al., 2017a). The G protein of rhabdoviruses is mainly involved in the assembly of virus particles and directly interacting with the M protein, promoting efficient virus budding (Sun et al., 2018). The G protein can also bind to host cell receptors, indicating that the G protein may be related to vector transmission (Eckert and Kim, 2001). The P7 protein of RSMV is predicted to be the L protein, which is a component of the viral nucleocapsid and contains the conserved main motif (GDNQ enzyme activity center) of the virus RdRp. The L protein is mainly involved in the catalysis of processes, such as capping the 5ꞌ end of mRNA, RNA binding, and polyadenylation (Ruedas and Perrault, 2009). P3 and P6 of RSMV are predicted to be non-structural proteins, and P3 has an NLS signal (Yang et al., 2017a). Studies have found that corresponding positions in the ADV, LNYV, rice yellow stunt virus (RYSV), and SYNV genomes encode movement proteins (Mann et al., 2016; Zhou et al., 2019a), but they share a low amino acid sequence identity with RSMV P3. Research has found that RSMV P3 can complement the cell-to-cell movement of PVX-GFPΔp25 and ToMV-GFPΔMP when expressed in trans, indicating that RSMV P3 may be a movement protein (Zhou et al., 2019a). The P6 protein of RSMV has a similar transmembrane region to the P6 protein of ADV and the P9 protein of BYSMV (Bejerman et al., 2015; Yan et al., 2015), so it may also be located in the cell membrane and involved in transmembrane transport. Moreover, the P6 protein of RYSV has been identified as a systemic RNA silencing suppressor and can form part of the viral structural protein (Guo et al., 2013). Taken together, the proteins encoded by plant rhabdoviruses have many functions, and future research will help to gain more knowledge about more functions of RSMV encoded proteins.

**Table 1:** Information on the putative viral proteins of RSMV^*^.

Protein name	ORF position (nt)	Gene size (bp)	Molecular weight (kD)	Putative function
P1/N	90–1,565	1,476	54.4	Nucleocapsid, N
P2/P	1,677–2,804	1,128	41.9	Phosphoprotein, P
P3/MP	3,014–3,547	534	20.1	Cell to cell movement
P4/M	3,738–4,262	525	19.6	Silencing suppressor, Matrix protein, M
P5/G	4,384–5,994	1,611	60.1	Glycoprotein, G
P6	6,013–6,213	201	8	Unknown
P7/L	6,286–12,486	6,201	235.9	Large subunit of polymerase, L

* Modified from Yang et al. (2017a).

## Rsmv Transcription and Replication

Rice stripe mosaic virus is a cytorhabdovirus and its transcription and replication occur in the cytoplasm. Studies have shown that animal and plant rhabdoviruses share many similarities in mRNA transcription and nucleocapsid replication. Based on studies of Vesicular Stomatitis Virus (VSV) and SYNV (Wagner and Jackson, 1997; Jackson, 2005; Jackson and Li, 2016), we propose the following transcription and replication process for RSMV (Figure 3B). After RSMV infects host cells, the viral gRNA serves as a template. The N-protein mRNA is the first transcribed mRNA, and the remaining mRNAs are transcribed sequentially in order of their appearance in the template *via* a stop–start mechanism, in which synthesis of each upstream mRNA is terminated before the transcription of the next downstream mRNA begins. This stop–start mechanism is repeated at each downstream gene–junction site to provide an elegant mechanism for polar mRNA transcription to produce decreasing amounts of N > P > P3 > M > G > P6 > L mRNAs (Whelan et al., 2004; Jackson and Li, 2016). The mRNA generated by the transcription is then used to translate and synthesize the seven viral proteins. As the core proteins (N, P, and L) gradually increase in abundance, the replication of gRNA and antigenomic RNA (agRNA) is promoted. In this process, the agRNA is wrapped by the N protein and combined with P and L proteins to form antigenomic nucleocapsids (agNCs), which promote agRNA replication to form gRNA (Whelan et al., 2004). Since the promoter activity of agNCs is stronger than that of genomic nucleocapsids (gNCs), the concentration of gRNA formed by replication is much higher than that of agRNA. The replication process consumes a large amount of N, P, and L proteins, which promotes the newly synthesized gRNA as a template to generate more viral mRNA (Banerjee, 1987) and causes the transcription and replication processes to alternately cycle.

## Disease Diagnosis and Detection

As mentioned above, RSMV generally induces slight dwarfing, mosaicism, and the twisting of leaves in host plants, and these symptoms can be used to diagnose RSMV in the field. However, some varieties of infected RSMV have similar symptoms to physiological stress caused by improper water and fertilizer management and the diagnosis is not accurate. Therefore, molecular diagnostics for RSMV detection and diagnosis based on reverse transcription (RT)-PCR have been developed in China (Chen et al., 2019a). Meanwhile, Guo et al. (2020) used virus particles as an immunogen to produce four RSMV-specific monoclonal antibodies (MAbs) for detecting the virus in field plants and vector insects. With these MAbs, RSMV can be readily detected in tissue crude extracts of RSMV-infected rice plants (Guo et al., 2020).

## Disease Cycle and Control

### Disease Cycle

**Figure 4:**
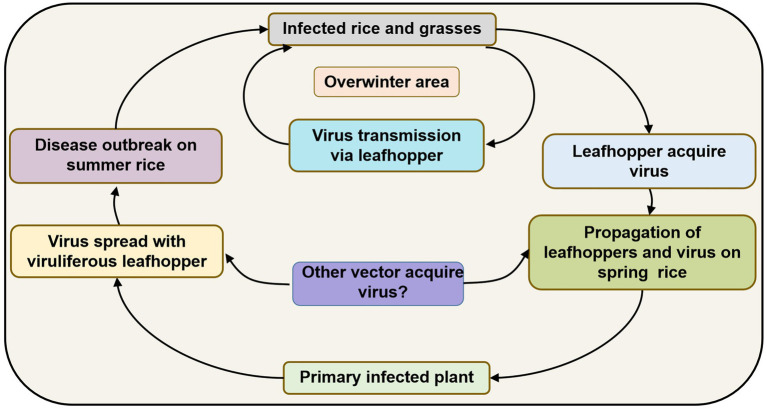
Infection cycle of rice stripe mosaic disease (RSMD) summarized based on Chen et al. (2019a).

Since *R. dorsalis* cannot migrate over long distances, overwintering plays an important role in carrying the virus among hosts from one season to the next. It may infect rice plants at low levels in spring and early summer, but infection gradually increases after several propagations and finally causes heavy epidemics and yield losses. Based on previous studies, the disease cycle of RSMD can be described as follows (Figure 4): RSMV and its vector *R. dorsalis* overwinter in rice as well as in some gramineous weeds such as *D. sanguinalis*, *M. vaginalis*, *E. indica*, and *A. aequalis*. In early spring, the overwintered nymphs acquire the virus by feeding on infected overwintered host plants. They then transmit the virus to new rice seedlings, causing primary infection. The 2–3 generations of leafhoppers that propagate in rice fields become viruliferous and spread the virus, causing reinfection. After the propagation of 3–4 generations, the newly hatched nymphs become viruliferous and then disperse into other fields, causing new disease outbreaks. Before and after the harvesting of summer rice, nymphs migrate to rice and some gramineous weed species for overwintering and can then transmit the virus to these hosts again, thereby completing the infection cycle (Figure 4).

### Disease Control

Many studies on the prevention and control of insect-transmitted plant viral diseases have been reported (Makkouk and Kumari, 2009; Zhou et al., 2013). Comprehensive prevention and control are the most common and effective measures in rice areas severely affected by viruses, including monitoring and accurate forecasting, screening resistant rice varieties, improving cultivation systems, covering rice seedling nurseries with insect-proof nets, and using pesticides rationally.

#### Monitoring and Accurate Forecasting

Field surveys of the disease and vector populations in early spring and summer rice can provide useful forecasting information, allowing farmers to undertake related control measures as soon as possible.

#### Screening Resistant Rice Varieties

Researchers have revealed that the cultivation of rice varieties with the RSV-resistant gene stv-b’ in Japan since the 1980s effectively controls RSV (Kisimoto and Yamada, 1986). However, work on screening and breeding rice cultivars with RSMV resistance is still ongoing. Therefore, in RSMV endemic areas, resistant and high-yielding varieties should be planted to reduce yield loss.

#### Improving Cultivation Systems

Adjustments to the planting period and the optimization of field management can affect the *R. dorsalis* life cycle to control RSMV epidemics. Researchers found that plowing after rice harvesting significantly reduces the population density of *Nephotettix cincticeps* and thereby decreases the incidence of rice tungro spherical virus (RTSV; Hirao and Ho, 1987). Therefore, plowing, drying, and irrigating the field after harvest or planting other crops in winter can prevent the disease from spreading widely by reducing overwintering and summer *R. dorsalis* populations. In addition, the seedling planting and sowing times should be properly chosen based on the *R. dorsalis* population dynamics. Basically, the sowing time should be delayed by 5–7 days so that the first generation of *R. dorsalis* does not coincide with the susceptible growth period of rice. For example, the rice planting time was delayed to avoid the migration peak of *N. cincticeps* and to reduce the incidence of RYSV in Guangdong, Guangxi, Hunan, and other provinces in China (Xie and Lin, 1984). Meanwhile, infected seedlings should also be diagnosed and replaced with healthy plants during early rice growth stages, and severely diseased fields should be abandoned and replanted with other crops (Zhou et al., 2013).

#### Covering Rice Seedling Nurseries With Insect-Proof Nets

Studies have shown that insect-proof nets are the most economical and effective preventive measure for rice viral diseases (Sun et al., 2013). Since they can prevent disease by blocking the transmission of *R. dorsalis*, the use of insect-proof nets to cover seedlings should be promoted in RSMV epidemic areas.

#### Use Pesticides Rationally

Pesticides should first be applied in rice fields to control overwintering or the first-generation of *R. dorsalis* and in rice seedling nurseries to reduce the *R. dorsalis* population and to thereby decrease transmission. Coating seedlings or transplanting seedlings with pesticides are recommended to control the *R. dorsalis* population in seedling nurseries.

## Epidemic Trends

Rice stripe mosaic virus was first discovered in Luoding, Guangdong province, in 2015. It is now widely distributed throughout southern China. For the following reasons, we speculate that RSMV will spread rapidly in the southern China rice region and surrounding countries in the future. First, climate change is likely to expand the overwintering region for RSMV and *R. dorsalis*, to increase the survival rate of the overwintering vector, and to thereby cause more serious and widespread RSMV outbreaks. Studies have found that *R. dorsalis* inhabits the mountainous and hilly rice growing areas of southern China and Southeast Asia. However, in recent years, *R. dorsalis* has also been found in the plains of rice growing areas, and it is thought that global climate change has led to the expansion of the *R. dorsalis* habitat, particularly to increases in its overwintering efficiency. The currently used rice cultivars and cropping systems also promote RSMD occurrence and lead to increased *R. dorsalis* populations in the field. In addition, the rice plants left behind by mechanical harvesting and the vigorous growth of ratoon rice seedlings provide a better habitat for *R. dorsalis* to survive. Last, the improper use of pesticides also promotes the expansion of *R. dorsalis*. For a long time, leafhoppers have not been regarded as important rice pests. However, the pesticides currently used are ineffective at controlling them and are harmful to their natural enemies, causing yearly increases in leafhopper populations in the field.

## Conclusion and Future Perspectives

This review described the most recent progress regarding the potential RSMV research directions and provides a more detailed understanding of this emerging virus, but near future research should focus on, but not be limited to, the following areas.

First, genetic resistance represents the most cost-effective option to improve and sustain crop yield in an environmentally friendly manner. Progress has been reported that different rice varieties exhibit slightly different symptoms and yield loss (Chen et al., 2019b). Therefore, identifying the RSMV resistance genes is worth to doing even though it could face the rapid emergence of virus resistance mutations.

Second, a better understanding of the interactions among the host-vector-RSMV is benefit to explore the management strategies of RSMV. A recent study found RSMV-infected rice plants can attract healthy *R. dorsalis* (Li et al., 2020), which has also been reported in other plant viruses (Mauck et al., 2010; Wang et al., 2013). Thus, comparative genomics, proteomics, and metabolomics can serve as powerful tools to identify host determinants involved in this phenomenon at the molecular level. Furthermore, insect vectors have always been the central role in the spread and outbreaks of plant virus disease. Besides *R. dorsalis*, *N. virescens* can also acquire the virus under laboratory conditions (Zhao et al., 2019). Therefore, identifying vector cell receptors and dissecting the vector transmission competency is a major obstacle. Meanwhile, whether there has other insect vector transmit RSMV in nature need to be further investigated.

Finally, negative-sense RNA plant virus have successfully developed reverse genetics system, which is increasing the understanding of viral biological functions, such as viral replication, vector transmission, and pathogenicity (Wang et al., 2015; Gao et al., 2019; Feng et al., 2020; Verchot et al., 2020). Hence, it is important to develop the RSMV reverse genetics system that will help to fully understand RSMV infection cycle, and may provide future tools for the management of this disease.

## Author Contributions

GZ and XY conceived and designed the conceptual structure of the manuscript. ZW, BC, and XY collected the literature and drafted the manuscript. TZ discussed and provided the critical suggestion. All authors contributed to the article and approved the submitted version.

## Conflict of Interest

The authors declare that the research was conducted in the absence of any commercial or financial relationships that could be construed as a potential conflict of interest.

## Publisher’s Note

All claims expressed in this article are solely those of the authors and do not necessarily represent those of their affiliated organizations, or those of the publisher, the editors and the reviewers. Any product that may be evaluated in this article, or claim that may be made by its manufacturer, is not guaranteed or endorsed by the publisher.
